# Prevalence of thyroid disorders in inland and coastal populations: A systematic review

**DOI:** 10.3389/fendo.2026.1811980

**Published:** 2026-07-21

**Authors:** Samyuktha Thakadiyil Harikrishnan, Amina Azeez, Sneha Reji, Daya Mani Jacob, Mustafa Sarmad Al Hamdani, Sondous Eksheir, Latifa Nabeel Alsaad, Jayakumary Muttappallymyalil, Satish Chandrasekhar Nair, Jayadevan Sreedharan

**Affiliations:** 1 College of Medicine, Gulf Medical University, Ajman, United Arab Emirates; 2 Dear Health Medical Center, Ajman, United Arab Emirates; 3 Department of Family & Community Medicine and Behavioral Sciences, College of Medicine, University of Sharjah, Sharjah, United Arab Emirates; 4 College of Health Sciences, Gulf Medical University, Ajman, United Arab Emirates; 5 Mohammed Bin Rashid University of Medicine and Health Sciences, Dubai Health, Dubai, United Arab Emirates; 6 Department of Community Medicine, College of Medicine, Gulf Medical University, Ajman, United Arab Emirates; 7 Thumbay Institute of Population Health, Gulf Medical University, Ajman, United Arab Emirates; 8 Abu Dhabi Healthcare Company SEHA-Pure Health Group, Abu Dhabi, United Arab Emirates

**Keywords:** deficiency, epidemiology, geography, goiter, hypothyroidism, iodine

## Abstract

**Background:**

Endocrine conditions such as thyroid disorders can significantly impact maternal and child health. Key determinants such as environmental setting and presence of iodine can influence the incidence of thyroid disorders. Individuals living in coastal regions are exposed to iodine rich foods in comparison to those residing in inland areas, which is a possible differentiating factor in the occurrence of thyroid based diseases in these individuals. Despite this possible relationship, very few studies have investigated the burden of thyroid diseases amongst coastal and inland populations.

**Objective:**

The study aims to systematically explore the prevalence of thyroid disorders in coastal and inland regions to understand the impact of differences in diet, environmental determinants, demographic variation and access to healthcare services on thyroid disease occurrence.

**Methods:**

Databases such as PubMed, Google Scholar and Scopus were used to identify articles in accordance with the PRISMA 2020 guidelines. Twenty three articles were selected based on them being in the English language, full length studies published between January 2017 and September 2023 and had reported on prevalence of thyroid diseases in inland and/or coastal areas.

**Results:**

The review identified that a higher prevalence of thyroid cancer and thyroid nodules were seen amongst coastal populations, while goiter and hypothyroidism were seen in inland and mountainous areas. These differences were influenced by the iodine status of the region, consumption of seafood, presence of environmental pollutants like bisphenol A and polychlorinated biphenyls, iodization of salt, socioeconomic conditions and healthcare access. Female gender and older age was found to be significantly associated with greater burden of disease in both areas of study.

**Conclusion:**

The geographical location or setting is a vital determinant of the distribution of thyroid disorders, mainly through the influence of iodine intake and exposure to environmental pollutants. Regular monitoring of environmental pollutants, early detection of cases, universal iodization of salt and proactive supplementation of iodine may lead to decreased prevalence of thyroid disorders and its consequences. Longitudinal studies in this field can help identify the causal pathways emerging from socioeconomic factors and environmental determinants to thyroid disorders.

## 1 Introduction

Hormones are important for the optimum functioning of the human body, thyroid hormones in particular are essential for development and growth of vital organs, reproduction and the regulation of metabolism, with extreme levels of the thyroid hormones causing clinical complications. Some common thyroid disorders include hypothyroidism and hyperthyroidism, which are mainly affected by determinants such as intake of iodine, genetic predisposition, chemicals, age, smoking and some therapeutic agents such as immune checkpoint inhibitors. Although thyroid cancer is seen to have low mortality, it is one of the fastest growing diseases affecting people worldwide (1). The GLOBOCAN database which records thyroid cancer cases amongst 185 countries in 2020, presented an age-standardized incidence of 10.1 per 100,000 women and 3.1 per 100,000 men. Fortunately, the age-standardized mortality rate was comparably lower with 0.5 per 100,000 women and 0.3 per 100,000 men for thyroid cancer. The incidence rates among women had high variation across different regions, whereas the mortality had remained the same, this signifies the important impact of environmental determinants on the identified burden of thyroid disease (2).

Iodine is known as the most important factor contributing to good thyroid health as it is a part of the synthesis of the thyroid hormone, with extreme levels of iodine causing abnormalities in thyroid function. Deficiencies in iodine is a public health issue combated by universal salt iodization (USI) programs, which has led to improved population health outcomes over the past few decades (3, 4). With time, populations are moving towards iodine sufficiency, creating shifts in thyroid disease patterns and differences in the prevalence of thyroid cancer subtypes to less aggressive forms. Therefore, ensuring the uptake of optimum levels of iodine has become a standard in public health measures across countries.

Other than iodine, thyroid disrupting chemicals (TDCs) have been a cause for concern in the environment. Chemicals such as bisphenol A (BPA) and polychlorinated biphenyls (PCBs) can affect thyroid function and promote autoimmune thyroiditis. These compounds have been the major cause for hypothyroidism in environments with sufficient iodine. High levels of these compounds collect into marine animals, which makes seafood consumption result in both high iodine intake and high exposure to TDCs. This double effect is one of the reasons why it is significant to compare thyroid diseases amongst coastal and inland communities and understand any underlying differentiating factors (5). An ecological study performed in Canada identified a skewed distribution of hypothyroidism in the coastal communities of Newfoundland, which may be due to the thyroid disease risk being affected by the proximity to coasts (6). Although such studies suggest a relationship between geography and incidence of thyroid diseases, the evidence is scattered, heterogeneous and locally based without systematic compilation.

It is vital to understand how the epidemiology of thyroid diseases can be influenced by inland and coastal environments, so that it can inform public health policy and clinical treatment guidelines for thyroid disorders. This systematic review was developed to summarize and critically evaluate scientific literature on the burden of benign and malignant thyroid disorders in both inland and coastal populations. Additionally, the review explores socioeconomic, dietary, environmental determinants that can impact the distribution and frequency of thyroid disorders. The systematic review aimed to find the prevalence of thyroid disorders and how it differs between inland and coastal areas.

## 2 Materials and methods

### 2.1 Protocol and reporting

**Figure 1:**
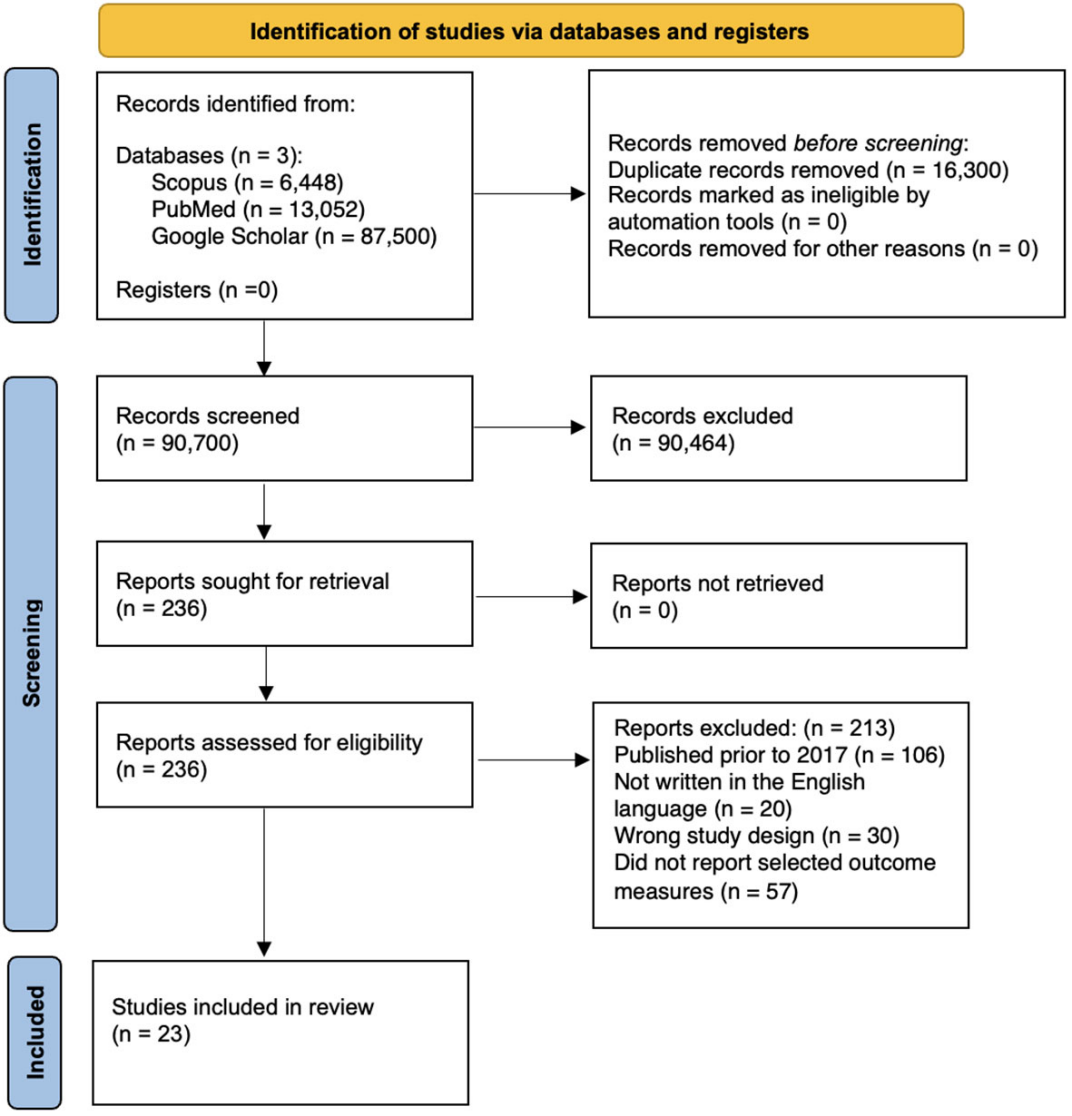
PRISMA 2020 flow diagram of the study selection process (33).

The Preferred Reporting Items for Systematic Reviews and Meta-Analyses or PRISMA 2020 guidelines were used to conduct this systematic review. The criteria and procedure for selection of articles and the number of studies included are presented in the PRISMA flow diagram below (**Figure 1**).

### 2.2 Eligibility criteria

The studies were mainly selected if it presented evidence on any thyroid disorders amongst inland and/or coastal populations and reported quantitative data and findings that can be used for comparison. Additionally, the studies were eligible if they were full length articles written in the English language and published between 1st January 2017 and 1st September 2023, the English language. The publication date period was selected to cover recent developments in iodine supplementation policies, environmental pollutants data and diagnostic practices to maintain a strong evidence base for analysis. Scientific research articles were excluded if they were in a non-English language, lacked data on thyroid prevalence, were not full length articles and if the articles were published before January 1st 2017.

The outcomes considered from the articles included both benign thyroid disorders such as hypothyroidism, thyroid nodules, hyperthyroidism, goiter and thyroid autoimmunity and malignant thyroid diseases such as thyroid cancer. When considering PICOS terms, the populations were from general populations or from inland and coastal regions, the exposure of interest was the geographical setting which could have been inland/coastal/urban/rural wherever specified, comparators were groups from inland and coastal regions, the outcomes were the epidemiological measures of thyroid disease such as prevalence of incidence and other iodine based indicators and study designs included in the review are observational studies such as cross sectional, case control and retrospective cohort studies.

### 2.3 Search strategy

A literature search was done systematically across three databases being PubMed, Scopus and Google Scholar which covered peer reviewed scientific articles from January 2017 to September 2023. The searches included key words related to geography, thyroid outcomes and iodine based terms. The keywords used for the literature search were (“geographical influence”, “thyroid abnormalities”, “thyroid nodules”, “goiter”, “coast” and “iodine”) using two Boolean AND and OR operators. Several search strings were used across databases which included the following: geographical influence AND goiter; geographical influence AND thyroid abnormalities; geographical influence AND thyroid nodules; coast AND goiter; coast AND thyroid abnormalities; coast AND thyroid nodules; iodine AND thyroid abnormalities; iodine AND thyroid nodules; and iodine AND goiter. The complete search strategy that was utilized in the three databases is provided in the **Supplementary File** (**Supplementary Table 1**).

### 2.4 Study selection

The articles from the three databases were compiled with duplicates removed. The first screening was based on the titles and abstracts being aligned with the eligibility criteria. The full text for the screened articles were then assessed individually by the authors to ensure inclusion for the review. The articles were selected through the process of identification, screening and inclusion as depicted in **Figure 1**. The references (1–9) are additional sources of scientific evidence that are cited in the Introduction (Section 1) and Materials and Methods (Section 2) to provide context and references (10–32) are the final selected studies included in the systematic review (as shown in **Figure 1**).

### 2.5 Data extraction

The included articles were used to extract specific data by the authors and compiled into a standard spreadsheet using excel. The extracted details involved bibliography based information such as author names, year and journal of publication, setting of the study, study design, characteristics of the population or participants included, thyroid outcomes observed, status of iodine, risk factors and other relevant findings that were important for the objective of the study. The information was then cross checked to ensure accuracy and consistency amongst studies. Screening of the studies based on title and abstracts were performed by four reviewers and any disagreements were discussed in detail and decisions were taken accordingly.

### 2.6 Outcome variables and operational definitions

The main outcome of interest was the prevalence of any thyroid disorder and its distribution and patterns presented in coastal and inland areas. This review relied primarily on the observational studies selected for review, therefore the classification of thyroid disorders followed the definitions used by each study. For example, hypothyroidism and hyperthyroidism were defined by the differentiating levels of serum thyroid- stimulating hormone (TSH) or free thyroxine concentrations, neck ultrasonography or palpation was used to identify thyroid nodules and goiter, and thyroid cancer was confirmed through histopathology.

The status of iodine was primarily assessed using the urinary iodine concentration (UIC) mentioned in the studies. According to the World Health Organization, a population which has median UIC levels below 100 µg/L has insufficient intake, 100 -199 µg/L reflects normal intake, 200 -299 µg/L is iodine above the requirements and ≥300 µg/L shows excess iodine intake (7–9). Other variables reported in the review pertained to use of iodized salt, consumption of seafood, environmental contaminants such as bisphenol A and general accessibility and affordability based conditions.

### 2.7 Quality appraisal and data synthesis

The included studies in this review are primarily cross sectional and based on other observational study designs. The heterogeneity present in this set of articles are addressed through a systematic review instead of a meta-analysis, wherein common determinants and risk factors were identified through comprehension and comparison across outcomes and features of each study. The 23 articles assessed for this systematic review is cited as references (10–32).

Risk of bias was assessed using the Joanna Briggs Institute (JBI) critical appraisal checklist for analytical cross-sectional studies (shown in **Table 1**). Each study was evaluated across eight domains: clarity of inclusion criteria, description of study subjects and setting, validity and reliability of exposure measurement, use of objective criteria for measuring the condition, identification of confounding factors, strategies to address confounding, validity and reliability of outcome measurement, and appropriateness of statistical analysis. Each domain was rated as Yes, No, Unclear, or Not applicable. Studies with all applicable domains rated “Yes” were considered low risk of bias, while studies with one or more domains rated “No,” “Unclear,” or “Not applicable” were considered medium risk of bias.

**Table 1:** Risk of bias assessment for included articles.

Study	Q1	Q2	Q3	Q4	Q5	Q6	Q7	Q8	Overall risk
Aktas and Demir, 2023 (10)									Medium
Al-Hakami et al., 2020 (11)									Medium
Chen et al., 2017 (12)									Low
Devaraj TV Salina S (13)									Medium
Dong et al., 2020 (14)									Low
Fan et al., 2021 (15)									Low
George et al., 2023 (16)									Medium
Guo et al., 2021 (17)									Medium
Krishna, 2021 (18)									Medium
Liu et al., 2021 (19)									Low
Liu et al., 2021 (20)									Medium
Lou et al., 2020 (21)									Medium
Lou et al., 2020 (22)									Low
Ramesh MK, 2017 (23)									Medium
Pal et al., 2018 (24)									Medium
Reddy et al., 2019 (25)									Medium
Shetty et al., 2019 (26)									Medium
Wang et al., 2020 (27)									Medium
Wang et al., 2021 (28)									Low
Wang et al., 2022 (29)									Low
Yan et al., 2021 (30)									Low
Zhu et al., 2022 (31)									Medium
von Oettingen et al., 2017 (32)									Medium

Yes, Green; No, Red; Maybe/Not Applicable, Amber/Yellow.

## 3 Results

### 3.1 Study selection and characteristics of included studies

The three databases identified 107,000 records from which duplicates were removed, this resulted in discarding 16,300 records. The screening of the title and abstract led to the exclusion of another 90,464 records. Next, the full texts of 236 articles were investigated for eligibility using the inclusion criteria, resulting in a final set of 23 studies to be included in the review (**Figure 1**). The studies were not constrained by geographical barriers, therefore it belonged to a diverse group of countries including India, Haiti, Saudi Arabia, Türkiye and China. The majority of the studies included were cross sectional in study design. The primary findings of the included studies [cited as references (10–32)] and its relevant observations and implications are recorded in **Table 2**.

**Table 2:** Comparative risk factors of thyroid diseases in inland and coastal populations.

Article no.	Study (first author, year, country)	Inland findings	Coastal findings	Common/shared risk factors
1	Wang X et al. (2021), China (28)	Hypothyroidism (subclinical and clinical) prevalent; higher median urinary iodine concentration (UIC); TPOAb positive	Access to iodized salt; Higher Income and educational level; Thyroid nodules prevalent; Metabolism syndrome	Older age; Female gender; Smoking; High BMI; Diabetes; Inadequate iodine intake; higher screening uptake for thyroid diseases
2	Lou X et al. (2020), China (21)	—	Higher median urinary iodine concentration (UIC)	Neonates with elevated TSH; Greater iodine concentration in drinking water; Lower intake of iodized salt
3	Zhu Y et al. (2022), China (31)	Not applicable	High prevalence of thyroid nodules	Older age; Female gender; High BMI; Living in eastern, northern, and northeastern regions; Higher concentration of iodine in drinking water
4	Fan L et al. (2021), China (15)	Not applicable	Increased thyroid cancer incidence	Low iodine intake; Areas with high goiter prevalence in children; Reproductive-age females; Greater frequency of medical examinations; High economic level
5	Devaraj & Salina (2021), India (13)	Not applicable	Greater anti-TPO antibodies; Greater prevalence of hypothyroidism	Female gender; Iodine deficiency; Thyroid autoimmunity is a risk factor for hypothyroidism
6	Lou X et al. (2020), China (22)	Not applicable	Not applicable	No intake of iodized salt; Low UIC; Female gender; Older age; Low educational level; engaging in agricultural work; Hypertensive patients; High BMI; Higher FBG, OTGG-2h, HbA1c, LDL, and TGAb levels; Lower TSH levels
7	von Oettingen et al. (2017), Haiti (32)	Mild iodine deficiency found in the mountainous region of Haiti (due to limited iodization initiatives)	Iodine sufficiency in coastal regions.	Pregnant and lactating women require more iodine, leading to iodine deficiency; Current breastfeeding, region and exposure to two endocrine environmental disruptors namely perchlorate and thiocyanate had a significant association to the competitive inhibition of iodine uptake, resulting in high urinary iodide levels.
8	Yan Y et al. (2021), China (30)	Not applicable	Not applicable	The study provided insights into the differences between urban and rural populations; Higher coverage of iodized salts seen amongst rural residents; Higher seafood intake, iodine-based drug use, and iodine contrast medium injection in urban areas; Older age may be a risk factor for thyroid nodules.
9	Al-Hakami et al. (2020), Saudi Arabia (11)	Not applicable	Not applicable	Presence of solitary nodules, MNG, large nodule size, and female gender increased risk of follicular carcinoma and other malignancies; Females above 45 years had higher prevalence of thyroid cancer than men.
10	Reddy TS et al. (2019), India (25)	Not applicable	No significant association between goiter and UIC, salt iodine levels or salt usage practices.	Consumption of iodized salt by women is likely to influence the prevalence of goiter; sufficient UIC observed despite goiter
11	Shetty A et al. (2019), India (26)	—	Increase the risk of thyroid cancer, possibly due to lower iodine nutrition; School aged children found with goiter prevalence, although many with sufficient UIC. Women affected more.	Exposure to goitrogen, High annual income, low iodine intake can increase risk of thyroid cancer; Low uptake of qualified iodized salt (CRQIS) and goiter prevalence (GP) increased thyroid cancer incidence.
12	Liu M et al. (2021), China (19)	Residents had lower body burden of mineral elements.	Residents had a higher body burden of mineral elements.	This study investigated the link between mineral element exposure and thyroid tumors and goiter. Exposure to certain mineral elements such as chromium, manganese, nickel, cadmium, antimony and thallium were found to be potential risk factors for thyroid tumors and goiter. Cadmium, mercury and thallium can also interfere with the balance of thyroid function (case control study).
13	Wang L et al. (2022), China (29)*	Not applicable	Greater prevalence of nodules present in urban coastal areas in comparison to rural coastal areas. Goiter is more prevalent amongst women.	Differences in urban and rural areas due to stress, environment or diet; <3 per week intake of fruit, dairy and vegetables associated with thyroid nodules.
14	Pal N et al. (2018), India (24)	Higher median UIC	Coastal rural areas (CRA) > coastal urban area (CUA) for median urinary iodine concentration.	Iodine status in mothers predicts sufficiency in iodine levels in infants with lactation creating higher requirements of iodine. Female gender; Older age; Consumption of vegetables and fruit < 3 times per week; Consumption of dairy products < 3 times per week; Occupation - students, housewives, employees of any enterprise/institution; Mental Health
15	Wang Z et al. (2020), China (27)	Not applicable	Presence of TPOAb; Subclinical Hypothyroidism	Higher need for iodine due to eating out, third-trimester pregnancy and non-iodized salt
16	Karthika & Ramesh (2017), India (23)	Low access to seafood; non-iodized salt	Greater iodine uptake; Variable iodine content in seafood	Lack of significant differences in TSH levels between coastal and noncoastal residents from the same panchayat; Misconception about iodine intake; Inconsistent use of iodized salt; Economic and educational challenges
17	Krishna B et al. (2021), India (18)	Not applicable	Influence of Geographical Location; Mean TSH levels slightly higher	Female gender; Older Age; Euthyroidism; Persistence of thyroid disorders despite iodine prophylaxis (through salt iodization program); coronary risk related to hypothyroidism (TSH > 10 mIU/L).
18	Liu X et al. (2021), China (20)	Compared to rural and mixed areas, greater nodule morbidity observed in urban areas.	In high altitude and coastal areas, there was greater thyroid nodule morbidity.	Female Gender; Older Age; Urbanization; Abnormal levels of iodine (both extremes)
19	George PS et al. (2023), India (Kerala) (16)	In midland and highland areas, there was lower incidence of thyroid cancer	Incidence of thyroid cancer collected in areas that are coastal and having low elevation (<20 m); mostly cases of papillary carcinoma.	Exposure to radiation; diet differences, residence in coastal regions; iodine consumption.
20	Aktaş & Demir (2023), Türkiye (10)	—	Ultrasound field study conducted in Central Black Sea region (Tokat)	Thyroid nodules and goiter seen to be correlated with parenchymal heterogeneity; female gender, obesity; association with older age.
21	Guo C et al. (2021), China (17)	Not applicable	Greater internal bisphenol exposures observed in children from coastal regions; Hyperthyroid children had higher levels of serum BPA in comparison to euthyroid children.	BPA is a modifiable risk factor; Inverse TSH-BPA correlation observed in euthyroid children with positive correlation amongst hyperthyroid children.
22	Chen C et al. (2017), China (12)	Not applicable	The coastal areas had environments with high iodine content. However, consuming non-iodized salt in these areas led to lower UIC levels and higher prevalence of iodine deficiency.	Non-iodized salt intake, female gender, and low UIC are risk factors for AITD.
23	Dong SY et al. (2020), China (14)	Not applicable	Central and lateral nodal metastasis observed in papillary thyroid carcinoma was correlated with BRAF V600E.	Considering individuals from coastal China, tumors without internal blood flow were seen to have mutation.

UIC, urinary iodine concentration; BPA, bisphenol A; TSH, thyroid-stimulating hormone; TPOAb, thyroid peroxidase antibody; TgAb, thyroglobulin antibody; BMI, body mass index; FBG, fasting blood glucose; HbA1c, glycated hemoglobin; LDL, low-density lipoprotein; AITD, autoimmune thyroid disease “—” signifies that information was not reported in the study. Quantitative estimates are presented in the table wherever reported.

*Article 13 (29) in **Table 2** is a preprint.

### 3.2 Modifiable risk factors

#### 3.2.1 Iodine intake and dietary patterns

The most prominent modifiable determinant appeared to be the status of iodine intake. Studies explained the possible relationship between coastal diets that have high proportions of sea food to a higher prevalence of thyroid nodules. In contrast, the inland communities that had iodized salt as part of their diets were associated with lower prevalence of nodules but greater prevalence of contracting goiter (28). Households based in the coastal regions utilized less iodized salt compared to the inland population, due to perceptions and beliefs that seafood rich in iodine is a safer food source of iodine (21). Increased incidence of thyroid cancer was associated with insufficient iodine intake with differences in iodine levels based on geography (15). Moreover, those consuming non iodized salt or seen to have abnormal urinary iodine concentrations were at risk to develop thyroid nodules (22). In contrast, individuals consuming greater iodized salt was associated with greater prevalence of thyroid antibodies such as Thyroid Peroxidase Antibody (TPOAb) and Thyroglobulin Antibody (TgAb), these proteins cause autoimmune disease by attacking the thyroid gland and the presence of these antibodies signify autoimmune diseases in individuals (12). Areas with low availability of iodine were seen to have more cases of goiter (10).

#### 3.2.2 Environmental pollutants

Many pollutants are known to enter the water based food chain, and remain a major contender in the cause of thyroid disorders. Higher serum bisphenol A or BPA concentrations were observed in children who had hyperthyroidism compared to children with euthyroidism. Similarly higher urinary BPA was recorded in middle aged and elderly people who had hyperthyroidism. The BPA concentrations were seen to correlate inversely with TSH concentrations, this supports BPA levels as a potential modifiable risk factor for thyroid diseases (17). A case control study investigating the impact of trace elements such as cadmium, thallium, antimony, nickel, chromium and manganese identified that mineral elements are potential factors contributing to thyroid cancer and goiter, with individuals residing in coastal areas having a greater presence of mineral elements in their body (19).

#### 3.2.3 Lifestyle and behavioral factors

Education, medical history of thyroid disorders, high consumption of seafood, and inadequate iodine levels were seen as potential risk factors leading to the development of thyroid nodules. Residing in coastal areas and in urban conditions were associated with a greater number or intensity of nodules when compared to living in interior areas (28).

### 3.3 Non-modifiable risk factors

#### 3.3.1 Age and sex

A few other risk factors identified in this review included older age and female gender in terms of non-modifiable risk factors for thyroid diseases. Females and older adults presented with greater predisposition to thyroid diseases such as presence of multiple nodules (17, 30). And individuals aged 45 years and above had a common occurrence of thyroid cancer (11). It is interesting to note that women during their reproductive period from 30 years to 50 years are observed to have higher burden of nodules, thyroid autoimmunity and goiter in comparison to men (10, 12, 30). Additionally, parenchymal heterogeneity and prevalence of nodules were seen to be significantly greater in females (10).

#### 3.3.2 Geographical setting

There are significant differences in the epidemiology of thyroid disorders when comparing individuals living in inland areas and those living in coastal areas. A higher prevalence of thyroid cancer and thyroid nodules was associated with living in coastal regions. In contrast, diseases such as goiter and hypothyroidism were seen more prominently in regions at high altitudes or based inland. The access to non-iodized salt in coastal areas presented with greater prevalence of thyroid abnormalities compared to inland areas (28). In Guangzhou, rural residents reported a higher prevalence of thyroid nodules and goiter in comparison to residents living in urban areas. In addition, goiter was associated with repeated pregnancies (30). Individuals living in urban, coastal or high altitude based conditions were seen to have higher nodule morbidity (20) with decrease in diary, vegetable and fruit consumption to be associated with nodules in coastal cities (29).

#### 3.3.3 Thyroid nodules and malignancy

Another non modifiable risk factor which is seen as a predictor of malignancy is the presence of thyroid nodules. Higher probability of follicular and various other carcinomas were associated with increase in nodule size, female gender, occurrence of solitary nodules and multinodular goiter (11). The prevalence of nodules is reduced with higher iodine consumption, which supports evidence stating that inadequate iodine is a risk factor for nodules. Moreover, nodules are more prevalent with increase in age, in plateau and coastal areas and with increased morbidity in females (20).

### 3.4 Combined coastal–inland variation in thyroid cancer

In Kerala’s Thiruvananthapuram district, the coastal belt had the highest cases of thyroid cancer in comparison to the inland areas, and a majority of the cases were found at low elevations (less than 20 meters); radiation exposure, iodine intake, and diet were identified as the main contributing factors. Most of the coastal adults were taking more iodine than required, and it was found to be associated with papillary thyroid carcinoma. This type of cancer was found to be present at a younger mean age in the coastal regions than in the midland or highland areas (16).

## 4 Discussion

Across the 23 observational studies, a generally consistent geographical pattern was revealed: thyroid nodules and thyroid cancer, in general, have been more frequently reported in coastal populations (20, 28, 29, 31), while goiter and hypothyroidism were more common inland and at high altitudes (20, 28). One might be inclined to interpret this difference as a direct influence of living near the sea, however, the data do not support such a simplistic deduction. Nearly all of the studies we included were cross-sectional or ecological, so they show associations at one point of time rather than causal chains, also, they differ greatly in how they defined coastal and inland, how they measured iodine status, and how they ascertained disease. A more plausible explanation is that geography per se is not a risk factor, but a marker of a combination of modifiable exposures (iodine intake and the behaviors that govern it, environmental contaminants, and unequal access to diagnosis) that happen to vary with location. Below we compare each pathway to the strength and consistency of the underlying evidence, and to the competing possibility that part of the observed geographical excess is a result of differences in disease detection and the extent of it.

### 4.1 Geographical patterns and the iodine paradox

The most noticeable aspect of coastal data is its internal contradiction. On the one hand, the consumption of seafood along with higher iodine in drinking water results in an increase in coastal iodine intake (21, 31); on the other hand, the same environmentally iodine-rich areas were reported as iodine-sufficient in some surveys (32) whereas in others they were classified as iodine-deficient. The review offers a very reasonable mechanism to reconcile the two, i.e. that coastal families, who are convinced that marine foods provide sufficient iodine, tend to use non-iodized salt more frequently resulting in a drop in measured urinary iodine even in places where dietary iodine is plentiful (12, 21, 27). This explanation of salt-behavior is very appealing because it not only moves the reason of explanation from a fixed feature, the coast, to a changed one, salt practice but it also must be regarded as a mere assumption rather than a confirmed discovery since none of the included studies has directly measured the salt-iodization behavior, dietary iodine and urinary iodine in the same persons with proper adjustment; the pathway was derived from the aggregate patterns rather than individual demonstration.

Some evidence is at odds with a strict coastal–inland dichotomy and deserves more influence than a purely confirmatory interpretation would give them. In one coastal population, besides inconsistent iodized-salt use and misconceptions about iodine, there was no statistically significant TSH difference between coastal and non-coastal residents of the same administrative area (23); whereas in the other, despite adequate urinary iodine, goiter was still a problem and was not significantly associated with salt iodine or salt usage (25). Such conflicting findings indicate that the coastal–inland comparison is often too simplistic, that variation in salt behavior and exposure to goitrogens within a region is usually greater than between-region variation, and that goiter can remain through mechanisms that are only partly reflected by one’s iodine status, for example, autoimmunity, goitrogen exposure or a history of deficiency. Another structural warning is that many geographical comparisons are ecological, i.e., they correlate regional disease rates with regional iodine indicators; therefore, deducing individual-level risk from them will lead to the ecological fallacy. Hence, the most prudent interpretation is the more limited one that the datasets actually back, namely, that local iodine behavior is much of a driver of the variation rather than coastal residence per se. This interpretation is in line with, though not confirmed by, earlier reports such as the uneven distribution of hypothyroidism in coastal Newfoundland (6).

### 4.2 Iodine status and dietary determinants

One of the clearest and most externally consistent signals in the review is the U-shaped relationship between iodine and thyroid disease (3) that the authors discovered: both insufficient iodine intake, which is a cause of goiter and is associated with a higher risk of thyroid-cancer incidence (10, 15), and excess iodine intake, which in some coastal areas has been associated with thyroid nodules and papillary carcinoma, were linked to the occurrence of harm. This dual pattern is both biologically logical and in agreement with the growing endocrinology literature. Besides, it is the result that the review can most confidently elaborate on.

Nevertheless, the causal explanation of each side varies significantly. The connection of iodine deficiency to goiter has long been recognized and is also directly supported by a physiological mechanism. The suggested connections between extreme iodine levels and cancer are much less solid: they mainly rely on a few cross-sectional or registry studies. Moreover, the link between high iodine and papillary carcinoma is not only Epidemiological but also challenging to disentangle from the detection bias or also ascertainment bias perspective because the increased use of neck ultrasound in iodine-sufficient high resource coastal areas mainly reveals the tiny papillary lesions (the ones resulting in carcinoma) that remain clinically silent for a long time. Thus, a seemingly increased incidence could be in part a reflection of intensified diagnosis rather than that of genuine biological risk. This point about diagnostic patterns is further elaborated in Section 4.5. What is much less susceptible to such bias is the well-established fact that maternal iodine status is a good predictor of infant iodine status, and that pregnancy and lactation are times of increased iodine requirements (21, 24, 27); and here, the iodine status or outcome is assessed directly via urinary iodine, hence the results were unaffected by how intensely the doctors searched for diseases. This maternal–infant axis is one of the review’s stronger points and also its most directly actionable.

### 4.3 Environmental pollutants

Several analyses have moved the conversation from just iodine to the thyroid-disrupting chemicals. The concentration of bisphenol inside bodies was more significant in children from coastal regions and it was related to changes in levels of thyroid hormones. High serum BPA was found in hyperthyroid kids and the BPA-TSH relationship was inverse (17). A case-control study blamed chromium, manganese, nickel, cadmium, antimony and thallium for thyroid tumors and goiter. The coastal individuals had a more significant load of these mineral elements in their bodies.

PCBs block the normal thyroid hormone transport and metabolism, while BPA can imitate thyroid hormone and can lead to disrupting the feedback regulation (28). Since fish is a common source of both, the beneficial iodine and the harmful contaminants, the coastal life may, on one hand protect from iodine deficiency and on the other hand expose to pollutants. This paradox calls for environmental monitoring and controlling fishing in addition to iodine programs.

### 4.4 Demographic determinants

Consistently across different studies and in various settings, being a woman and/or older age has been very much linked with getting thyroid disease (10–12, 14, 16, 30); however, this finding which repeated in populations with different geography, iodine status and design, is one of the review’s most firm conclusions. It can be best seen as a common underlying susceptibility on which environmental and behavioral factors play, and it allows a clear distinction of the relatively stable question of who is at risk from the more variable question of where and why.

Two caveats serve to clarify this interpretation rather than erasing it. Firstly, the female excess is probably real, in line with the established autoimmune and hormonal biology, and here it is further supported by the occurrence of autoimmune thyroid disease in coastal women with low urinary iodine (12, 17); but on the other hand, the size of the effect as measured is magnified to an unknown extent by sex-differential ascertainment, since women are tested for thyroid more often during antenatal and reproductive care. Secondly, associations with age reflect that more than cumulative biological risk, they reveal birth-cohort differences in iodine exposure or even the greater chance for incidental detection that increases with age. Demographic factors thus remain real and helpful for the purpose of screening, although their level should be interpreted with the same caution as the geographical one because of detection issues.

### 4.5 Socioeconomic status and healthcare access

Variations in socioeconomic status and healthcare access make it difficult to understand the geographical gradient. Several studies reported that the coastal populations were of a higher income and had better healthcare access, which results in increased detection rates and therefore the apparent prevalence of thyroid disorders, whereas the treatment is also available earlier with fewer complications (12, 15). On the other hand, the inland and the lower-resource areas had lower detection rates and limited facilities (13). A higher prevalence recorded in coastal areas may therefore partly be due to the surveillance and detection bias rather than a real excess of disease - a very significant point not to be forgotten when handing over these results to the policymakers.

### 4.6 Implications for clinical practice and public health

One should not consider all findings equally settled while making practical implications. The most powerful and regular evidence supports the simplest and least controversial actions: continuing and enforcing the universal salt iodization especially in iodine-deficient inland and high-altitude regions and giving targeted supplementation to pregnant and lactating women, both of which rely on strong, low-bias findings and directly tackle the goiter and hypothyroidism burden (3, 24, 27).

Public-health communication campaigns in coastal areas can help in changing the mindset of people that seafood can replace the need for using iodized salt. This is a low-cost and strongly justified measure which is in line with the salt-behavior hypothesis, although this hypothesis has not been proven yet. On the other hand, environmental monitoring and regulation of contaminant exposure (17, 19), while surely a sensible precautionary measure, are more likely to be framed as a response to a potential but not yet verified risk than an established requirement.

One of the consequences is that it contradicts the initial assumption that more screening is always better, and for that reason, this point deserves special attention as the detection-bias analysis makes it clear: increasing ultrasound screening in coastal populations might amplify the overdiagnosis of small, indolent papillary cancers that have led to an increase in prevalence figures in the first place, thereby possibly leading to overtreatment without sufficient benefit. Hence, the focus in terms of equality should not be on just more case-finding everywhere but on case-finding that is better-targeted: identifying and addressing diagnostic gaps for symptomatic and deficiency-related diseases in under-resourced areas besides avoiding low-value detection of lesions that are not likely to cause harm.

### 4.7 Strengths and limitations

The review scanned three main databases, concentrated on recent studies (2017-2023), and based on populations in parts of Asia, the Caribbean, and the Middle East, enabled a cross-disciplinary assessment of how dietary, environmental, demographic, and geographical factors influence thyroid disease along the entire range from minor disorder to cancer.

A few limitations are worth mentioning. Most of the evidence was based on cross-sectional studies, which can only show associations and not cause-and-effect relationships. Many of the studies were conducted in China, which presents a potential geographical bias and makes it harder to generalize the results. Different studies showed considerable differences in study design, population characteristics, definitions of coastal and inland areas, iodine assessment methods, and thyroid disorder diagnostic criteria, which not only prevented formal meta-analysis but also greatly weakened the comparative conclusions (15, 21, 25, 28, 31). Important confounders such as smoking status, body mass index, pregnancy, family history of thyroid disease, and medication use were not systematically reported across the studies, hence these factors could only partially be adjusted in this review (13, 16, 18, 28). Differences in health care access and screening levels between regions could have led to detection bias, i.e. higher incidence of thyroid disorders in coastal urban populations may simply be due to more diagnostic activities rather than reflecting a bigger problem (10, 15, 32). Meanwhile, regional differences in iodine policies, salt iodization programs, and their implementation level were very different across the studied countries, which again make it very difficult to compare directly (15, 20, 21). These limitations should be taken into account when interpreting the results of this review. Variations in study designs, populations, iodine-status assessment, and diagnostic criteria prevented meta-analysis and made direct comparison between studies difficult.

Variation in baseline iodine nutrition further limited comparability. Limiting to English-language articles only from the selected databases might have led to a language and publication bias, whereas the inclusion of a preprint only serves to show the necessity of verifying findings in peer-reviewed studies. These limitations somewhat weaken the conclusions and, on the other hand, suggest quite definite ways for future longitudinal and standardized research.

## 5 Conclusion

The differences that were noticed in how widespread thyroid issues are and their types in populations living by the sea versus those living inland seem to be related to a number of things, such as differing levels of iodine, eating of seafood, exposure to environmental pollutants, and disparities in healthcare access and screening methods. Nevertheless, since the majority of the studies considered were observational, it is not possible to determine cause-effect relationships. It is plausible that better-off coastal areas might identify more cases of the disease, so some of the apparent excess could be due to heightened surveillance. Enhancing universal salt iodization and providing iodine supplements selectively, along with environmental monitoring and better identification of cases, constitute an efficient way of decreasing the thyroid disease burden which is subject to change. More studies over time are necessary to unravel the cause-and-effect relationships between environmental and socioeconomic factors and thyroid health. Also, such a work would pave the way for an equitable public health policy for both inland and coastal communities.

## Data Availability

The original contributions presented in the study are included in the article/**Supplementary Material**. Further inquiries can be directed to the corresponding author.
