# Supplementary material for: Prevalence of thyroid disorders in inland and coastal populations: A systematic review
Source: Front Endocrinol (Lausanne). 2026 Jul 21;17:1811980. doi: 10.3389/fendo.2026.1811980 (PMC13433190; doi:10.3389/fendo.2026.1811980)
Supplement: Supplementary file 1 [file Table1.docx]

Supplementary Material

Table 1. Electronic Search Strategy (16^th^ October 2023)

| **Database** | **Search No.** | **Search String** | **Records Retrieved** |
| --- | --- | --- | --- |
| Scopus | S1 | geographical AND influence AND goiter | 706 |
|  | S2 | geographical AND influence AND thyroid AND abnormalities | 884 |
|  | S3 | coast AND goiter | 609 |
|  | S4 | coast AND thyroid AND abnormalities | 826 |
|  | S5 | coast AND thyroid AND nodules | 281 |
|  | S6 | thyroid AND geography | 3142 |
|  |  |  |  |
|  |  | **Total Scopus records that are identified** | **6,448** |
|  |  |  |  |
| PubMed | P1 | geographical AND influence AND goiter | 28 |
|  | P2 | geographical AND influence AND thyroid AND abnormalities | 7 |
|  | P3 | geographical AND influence AND thyroid AND nodules | 7 |
|  | P4 | coast AND goiter | 35 |
|  | P5 | coast AND thyroid AND abnormalities | 14 |
|  | P6 | iodine AND thyroid AND abnormalities | 1731 |
|  | P7 | iodine AND thyroid AND nodules | 1956 |
|  | P8 | iodine AND goiter | 9274 |
|  |  |  |  |
|  |  | **Total PubMed records that are identified** | **13,052** |
|  |  |  |  |
| Google Scholar | G1 | geographical AND influence AND goiter | 15,400 |
|  | G2 | geographical AND influence AND thyroid AND abnormalities | 18,600 |
|  | G3 | geographical AND influence AND thyroid AND nodules | 17,100 |
|  | G4 | coast AND goiter | 11,200 |
|  | G5 | coast AND thyroid AND abnormalities | 18,000 |
|  | G6 | coast AND thyroid AND nodules | 7,200 |
|  |  |  |  |
|  |  | **Total Google Scholar records that are identified** | **87,500** |
|  |  |  |  |
|  |  | **Total records identified from all databases** | **107,000** |
